# Unmet need for modern contraception and associated factors among reproductive age group women in Eritrean refugee camps, Tigray, north Ethiopia: a cross-sectional study

**DOI:** 10.1186/s13104-018-3956-7

**Published:** 2018-12-04

**Authors:** Kidane Gebrecherkos, Brhane Gebremariam, Abebaw Gebeyehu, Hailay Siyum, Gizienesh Kahsay, Mebrahtu Abay

**Affiliations:** 1 Department of Public Health, College of Health Sciences, Aksum University, Aksum, Ethiopia; 2 Institute of Public Health, College of Health Sciences, University of Gondar, Gondar, Ethiopia

**Keywords:** Unmet need, Contraceptive use, Reproductive age women, Refugee camp, Tigray, Ethiopia

## Abstract

**Objective:**

Millions of women want to delay or avoid pregnancy, but they are not using contraception, especially in refugee settings. Due to lack of contraception, one fifth of reproductive age group women suffered from unwanted pregnancy and unsafe abortion, which accounted for 78% of maternal mortality in refugee camps. Therefore, the aim of this study was to assess the prevalence of unmet need for modern contraception and its associated factors among reproductive age group women in Eritrean refugee camps, Tigray, Northern Ethiopia, 2016.

**Results:**

400 women of reproductive age group interviewed. Prevalence of unmet need for modern contraception in this study was found to be 41.8% (95% CI 36.99%, 46.63%).Respondents’ unfavorable attitude towards modern contraceptive methods [AOR = 0.372, 95% CI 0.170, 0.818] and the availability of modern contraceptive methods [AOR = 3.501, 95% CI 1.328, 9.231] were factors significantly associated with unmet need for modern contraception. Respondents’ attitude towards modern contraceptive methods and availability of modern contraceptives were independent predictors of unmet need. Governmental and non-governmental organizations should design programs to create behavioral change in women’s attitude towards contraceptive use and to secure the availability of contraceptive methods in refugee camp settings.

## Introduction

According to WHO, 2016 report unmet need for contraception is the proportion of currently married or in sexual union women desiring to limit or space childbearing but not using any contraceptive methods. Unmet need remains a useful tool in identifying and targeting women at high risk of unintended pregnancy. It is one of the indicators used for monitoring of family planning programs [1]. The use of modern contraceptive methods remains an important component in the reduction of fertility as well as maternal, infant and child morbidity and mortality [2, 3].

Many women in crises, be it social crisis like in refugee camps or economic crisis, are vulnerable from the insecurity and disruption of an emergency, they usually face a continuing risk of unwanted pregnancy and various maternal complications. Furthermore, they are at increased risk of sexual violence and its consequences [4]. UNHCR estimates that around 15.4 million border-crossing refugees found worldwide in 2011. Among those, females and children accounted for 48% and 46% of the total refugees respectively [4]. Ethiopia was the sixth largest country with regard to number of refugee population having a total of 376,400 refugees by the end of 2012 [5]. women and children in refugee camps may actually increase their risk of being sexually violated, and are exposed to rape, unintended pregnancy, unsafe abortions and spread of STI /HIV which lead them to long-term psychological problem and high infant & maternal mortality [6, 7].

According to UNHCR, 2015 health information system (HIS) annual report, the utilization of modern contraceptive methods among Eritrean women, reside in refugee camps of Tigray, Ethiopia is low (32%) [8]. Complications during pregnancy or childbirth are the leading causes of death and disability among women of reproductive age group in developing countries. This is even worse in areas where there is conflict as it leads to lack of accessibility to family planning [9].

Refugee women in conflict situations are at an increased risk for unintended pregnancies, poor child spacing, unfavorable pregnancy outcomes and STIs including HIV [10]. The higher rate of unmet need is worse among refugees in Sub-Saharan Africa. Rural residents, un-educated and poor women are also at higher risk [11–13].

Displaced women expose to sexual violence, sexual exploitation and abuse, which all lead to different health related complications [14, 15]. Over all, lack of contraception leads to impediments in health, human rights, social and economic development. These conditions indicate that reproductive health care is crucial need of refugees, from comprehensive family planning support to emergency contraception provision [16–18].

As there is no previous study in the study area, this study aims at assessing the prevalence and predictors of unmet need for modern contraception among the reproductive age group women of Eritrean refugee’s in Tigray, north Ethiopia.

## Main text

### Methods

#### Study setting, study population and sampling

Community based cross-sectional study was conducted among women of reproductive age group in Eritrean refugees found in Tigray, Ethiopia from July to September 2016. There are four Eritrean refugee camps in shire operation named as “*Adi*-*harush*”, “*Shimelba*”, “*May*-*ayni*” and “*Hitsats*”, which are located 1167, 1203, 1150, and 1120 Kilometers far from Addis Ababa, the capital of Ethiopia. According to UNHCR 2016 population report, there were 6680 reproductive age group women refugees in the camps. After we obtained a list of each household from the respective refugee, reproductive age group women were selected proportional from each refugee camp. Finally, systematic random sampling method was used to get respondents. Sample size was determined using a single population proportion formula with the assumptions prevalence of unmet need (p = 0.5, because there was no previous study), 95% CI (**Ζ**_1−α/2_) = 1.96), 5% degree of marginal error (d), finite population correction formula (because the total women in the refugee camps were < 10,000) and 10% non-response rate. The sample size was 400 [19].

#### Data collection and data quality control

Data were collected using structured interview administered questionnaire, which was adapted and modified from (EDHS), 2011 [20]. The questionnaire was pretested by the principal investigators in “*Endabaguna”* Town, Tigray, and north Ethiopia.

Data were collected by interviewing the respondents. Eight diploma nurses collected the data and supervised by four BSc nurses. To assure quality of the data, training was given to the data collectors and supervisors by the principal investigator for 2 days. The supervisors and principal investigator checked the filled questionnaires daily. During data collection, if there were more than eligible women in a household one woman selected randomly.

#### Variables and measurement

*Dependent variable* Unmet need for modern contraception (unmet versus met need).

*Independent variables* Socio-demographic, reproductive, contraceptive related and service provision related variables.

*Favorable attitude towards modern contraceptive methods* Respondents who scored points greater than or equal to the mean score (45.2) of the total 12 items of attitude related questions.

*Knowledgeable about modern contraceptive methods* When a women is able to mention at least one modern contraceptive methods by here self or know after description of any modern method.

*Unmet need for modern contraception* Refers to the contraceptive need of fecund and currently married women or women in union who are either not pregnant and want child later on or not at all, or who are pregnant as result of a miss-timed or unwanted pregnancy but not using any contraceptive method.

*Unmet need for limiting* The percentage of non-pregnant women who do not want another child at all and are pregnant because of unwanted pregnancy, but not using any modern contraceptive method.

*Unmet need for spacing* The percentage of not pregnant women who want another child after 2 years and are pregnant as result of a miss-timed pregnancy, but not using any modern contraceptive method.

#### Data management and analysis procedure

We coded, cleaned and entered the collected data by using SPSS version 21.0 and analyzed. Descriptive statistics like mean and standard deviation were used to quantitative data, and frequencies and proportions used for categorical data. Binary logistic regression analysis using odds ratios (ORs), 95% confidence intervals (CIs) were used to determine association between predictors and the outcome variable. Variables with the P-value of less than 0.05 in both the bivariable and multivariable logistic regression models were considered as statistically significant predictors.

### Results

A total of 400 participants were included in the study having a 100% response rate.

#### Socio-demographic characteristics

Data from 400 reproductive age group women were analyzed. The mean (± SD) age of the women was 28.39 (± 7.15). Related to the marital status and residency, 314 (78.5%) were married and 238 (59.5%) reside in rural areas before displaced from their country. Regarding ethnicity, 245 (61.3%) were Tigrigna. A little above half (54%) of the total respondents were attended elementary school and were Orthodox Christian (55.5%) by religion. More than two-third (69.3%) of the respondents were housewives by occupation before displacing. Three hundred forty five (86.3%) of partners/husbands of the women had attained formal education, and 315(78.8%) of them had occupation (Table 1).

**Table 1:** Socio-demographic characteristics of reproductive age group (15-49) women in Eritrean refugee camps in Tigray, north Ethiopia, 2016

Variables	Category	Number (n)	Percent (%)
Age of women (in years)	15–19	41	10.25
	20–24	84	21.00
	25–29	113	28.25
	30–34	83	20.75
	35 and above	79	19.75
Religion	Orthodox	222	55.50
	Muslim	84	21.00
	Protestants	38	9.50
	Catholic	56	14.00
Ethnicity	Tigrigna	245	61.25
	Saho	83	20.75
	Kunama	72	18.00
Marital status	Married	314	78.50
	Single	26	6.50
	Divorced	36	9.00
	Widowed	24	6.00
Educational status	Illiterate	78	19.50
	Elementary school (1–8th)	216	54.00
	Secondary school	93	23.25
	Higher education	13	3.25
Occupational status	House wife	277	69.25
	Daily laborer	82	20.50
	Student	41	10.25
Husband’s religion	Orthodox	240	60.00
	Muslim	74	18.50
	Protestant	30	7.50
	Catholic	56	14,00
Monthly household income (in Eriterian Nakfa)	< 400	45	11.25
	401–745	64	16.00
	746–1220	113	28.25
	> 1220	178	44.50

#### Reproductive health characteristics

The mean (± SD) age at first marriage was 19 (±  2.6) years old. Three hundred forty-three (85.8%) women had history of pregnancy, among those 349 (87.4%) had more than one pregnancy in their lifetime. A hundred and sixty-seven (41.8%), women had unmet need for modern contraception in the study. Three hundred sixteen (79%) respondents have ever heard about modern contraceptive methods. Out of those, 157 (39.25%) participants had knowledge and 235 (58.75%) had favorable attitude towards use of modern contraceptive methods. Two hundred forty-five (61.5%) of the women had discussed with their husband on modern contraceptive methods use and 182 (45.5%) of the respondents were supported to use contraceptive methods by their husbands. Both partners decided to use modern contraceptive methods was 203 (50.8%) and decided on the number of children to have for the future in their life together 291 (72.8%). Two hundred eight (52%) of the respondents had history of exposure to media about modern contraceptive methods and 192 (48%) had not (Table 2).

**Table 2:** Reproductive health characteristics of reproductive age group women in Eritrean refugee camps in Tigray, north Ethiopia, 2016

Variables	Category	Number (n)	Percent (%)
Age at first marriage(in years)	< 18	176	47.10
	≥ 18	198	52.90
History of abortion	No	279	74.60
	Yes	95	25.40
Desired number of children	≤ 5	179	47.86
	> 5	71	18.98
	Don’t want more child	64	17.11
	Undecided	60	16.04
Ever use of modern contraceptive methods	No	203	50.75
	Yes	197	49.25
Current use of modern contraceptive methods	No	296	74.00
	Yes	104	26.0
Types of modern contraceptive methods currently used	Oral pill	31	29.81
	Injectable	52	50.00
	Implant	15	14.42
	IUCD	4	3.85
Current pregnancy status among non-users	Non-pregnant	187	63.18
	Pregnant	109	36.82
Type of pregnancy	Wanted now	63	57.80
	Unwanted	11	10.09
	Mistimed	35	32.11
Current amenorrhea among non-users	Not amenorrheic	197	66.55
	Amenorrheic	99	33.45
Reason for not being pregnant	Wanted child later on	92	49.20
	No more want child	29	15.50
	Want child soon	54	28.88
	In fecund	12	6.42
Anti natal care (ANC) visit	No	13	11.93
	Yes	96	88.07
Age started using modern contraception methods	15–19	42	21.30
	20–24	107	54.30
Respondent’s knowledge about modern contraceptives	≥ 25	48	24.4
Respondent’s attitude towards modern contraceptive use	Knowledgeable	157	39.25
	Not knowledgeable	243	60.75
	Favorable attitude	235	58.75
	Unfavorable attitude	165	41.25

#### Prevalence and factors associated with unmet need for modern contraceptive methods

The total unmet need for modern contraception was 41.8% (95% CI 36.99%–46.63%) with 31.8% unmet need for spacing and 10% unmet need for and limiting. The analysis of bivariable logistic regression indicated that history of abortion, ever use of modern contraceptive methods, number of ANC visits and exposure to media about modern contraceptives were variables significantly associated with modern contraception. In addition, attitude towards modern contraceptives, discussion with husband on contraception, availability of modern contraceptive methods in the health facility, and discussion on contraception with community health workers were significant variables associated with unmet need for modern contraception with p-value < 0.05.

In the multivariable logistic regression model, respondents’ attitude towards modern contraceptive use and availability of modern contraceptive methods were significantly associated with the unmet need for modern contraceptive use. Women who had no enough availability of modern contraceptive methods were 3.5 times more likely to have unmet need for modern contraception as compared to those who have [AOR = 2.77 95% CI (1.63, 4.70)]. Women who had favorable attitude towards utilization of modern contraceptive methods were less likely to have unmet need for modern contraception by 63% as compared to those who had unfavorable attitude [AOR = 0.49 95% CI (0.31, 0.79)] (Table 3).

**Table 3:** Factors associated with unmet need for modern contraception methods among reproductive age women in Eritrean refugee camps, Tigray, north Ethiopia, 2016

Variables	Category	Unmet need		COR (95% CI)	AOR (95% CI)
		Yes	No		
		Number (%)	Number (%)		
Attitude towards contraceptive use	Favorable	72 (30.6)	163 (69.4)	0.33 (0.22, 0.49)*	0.49 (0.31, 0.79)**
	Unfavorable	95 (57.6)	70 (42.4)	1.00	1.00
Availability of modern contraceptive methods	Yes	98 (33.1)	198 (66.9)	1.00	1.00
	No	69 (66.3)	35 (33.7)	3.98 (2.48, 6.39)*	2.77 (1.63, 4.70)**

* Significant in the bivariable logistic regression at p-value < 0.05

** Significant in the multiple logistic regression at p-value < 0.05

### Discussion

The overall prevalence of unmet need for modern contraception was 41.8% (95% CI: 36.99%-46.63%). This is higher than studies conducted in Djibouti (8.8%), Kenya (9.9%), Uganda (7.8%) and Canada [21–24]. Whereas, it is lower than a study conducted in Uganda (52.1%) [25]. The discrepancy might be due to differences in expanded health service provision, availability and awareness to contraceptive methods, socio economic status, and educational status.

In multivariable analysis, respondents’ attitude towards modern contraceptive methods and availability of modern contraceptive methods found to be significant and determinant factor for the unmet need.

Women who had no enough availability of modern contraceptive methods were more likely to have unmet need as compared to those who have **[**AOR= 2.77, 95% CI (**1.63, 4.70)]**. This is consistent with the multi-country baseline assessment done among women selected refugee settings [26]. This result also supported with studies conducted among refugee women in Nigeria and Pakistan [27, 28]. Those studies suggested that there was a lack of availability to use modern contraceptive methods; none of the clients was using long and permanent modern contraceptive methods. At the same time, female condom utilization was nearly zero, emergency contraception was only available in the situation of post rape care; only short-term contraceptive methods reported. This causes low utilization of modern contraceptive services in the refugee settings.

Respondents’ attitude towards modern contraceptive use has a relevant input to meet the unmet need. Women who had favorable attitude towards utilization of modern contraceptive methods were less likely to have unmet need as compared to those who had unfavorable attitude **[**AOR =0.49, 95% CI (0.31, 0.79**].** This finding was supported by a research conducted among refugees in Ethiopia [19]. The possible explanation could be women having unfavorable attitude towards modern contraceptive use influence the utilization of the services.

### Conclusion

The prevalence of unmet need for modern contraceptive methods in the study area was 41.8%. Women attitude towards modern contraceptive methods and availability of modern contraceptive methods were significantly and independently predictors of unmet need for modern contraception.

Different stakeholders, together with Ethiopian government, should take a commitment to strengthen the availability of modern contraceptive supplies in the Eritrean refugee camps. They should also develop, together with health professionals, implementing programs to bring behavioral change and favorable attitude towards modern contraceptive use among Eritrean women in the refugee camps.

## Limitation

The study used self-report (using questionnaire) method to measure the different independent variables, which may sometimes result in biased result.

## Abbreviations

ARRA: administrative for refugees and returnee affairs
CSA: Central Statistical Agency
DHS: Demographic Health Survey
CPR: contraceptive prevalence rate
EDHS: Ethiopian Demographic Health Survey
FMOH: Federal Ministry of Health
FP: family planning
HIV: human immune deficiency virus
IDP: internally displaced persons
IRC: International Rescue Committee
NGO: Non-Governmental Organization
PI: principal investigator
SPSS: Statistical Package for the Social Sciences
STI: sexual transmitted infections
WHO: World Health Organization
UN: United Nations
USAID: United States Agency for International Development
UNFPA: United Nations Fund for Population Activities
UNHCR: United Nations Higher Commissioner for Refugees
